# Points in Extraction

**Published:** 1893-04

**Authors:** R. W. Gillett


ARTICLE II.

### POINTS IN EXTRACTION.

BY R. W. GILLETT.

**Extracts from a paper read before the Students' Society, Dental Hospital of London.**

Before commencing my paper I think a few introductory remarks would not be out of place, so that we can have a clear understanding as to the scope of the paper. The heading of my paper may be rather misleading to some of you, as it was decided upon before I had really given the subject my whole attention. I shall not attempt to describe to you the different methods of extracting the various teeth, as I believe that they may only be learned by constant practice in the extracting-room. I shall only make mention of a few general hints With regard to the instruments, little can be said dogmatically, as every one will probably have a different opinion.

We next proceed to examine the mouth, looking for: exposed nerves, especially chronically inflamed pulps; diseased roots; especially chronic periostitis and exostosis; irritation of the nerve from a filling or nearly exposed pulp or pulp-stones; impacted teeth, especially wisdoms. The wisdom teeth are the most common cause of neuralgia, as their roots are situated close upon the trunks of the nerves, and are sometimes perforated by them. Exposed nerves are best found by introducing a small plug of cotton-wool on a pair of dressing forceps, which usually gives pain in the region referred to. Diseased roots may be found by tapping the teeth one by one, and by the presence of inflammation in the gums. For exostitis the teeth should be pressed from side to side. Irritation of the pulp may best be found by applying to each tooth a piece of cotton-wool soaked in iced water. Impacted teeth are usually discovered from the absence of one of the teeth from the series, and signs of inflammation, especially swelling of the part.

THE EXTRACTION OF TEMPORARY TEETH.—With regard to the forceps, I think that the forceps we now use in the extracting-room for children's teeth are far from perfect. Why should we attempt to extract a child's temporary tooth with a pair of forceps which are meant for a fully grown man? Of course, temporary teeth can easily be extracted with almost any forceps, but are we extracting so as to give the least possible pain? I think children's forceps should be so small that they can be held in the operator's hand so as not to be seen by the child. Forceps should be made to fit as accurately as possible round the roots of the teeth to be extracted, with just sufficient room to clear the crown. I do not see why they should not be made after the same pattern as those for the permanent teeth, but they should be proportionately small. Temporary teeth as a rule, in a patient child, are not much trouble to extract, but when they have been retained in the jaws long after their time for removal, and their roots are unabsorbed, they will often cause some little trouble to extract, as their roots are very divergent and the alveolus has become very dense around them. Temporary teeth will often be found lying on the gum in a necrosed condition; their removal is then most easily accomplished by means of the elevator and forefinger. A pair of large dressing forceps with serrated blades are also very useful to remove such fragments. Care should be taken in the extraction of temporary teeth not to go up too far, as there is some danger of injuring the permanent successor. For various causes the extraction of a temporary tooth is often not at all advisable. We must, therefore, do something to relieve the pain. If it is caused by an exposed nerve, a dressing should be applied of two parts of creasote to one of mastic on a little cotton-wool; or a very good plan is to place a tabloid of cocaine over the exposure, and keep it in place with some cotton-wool and mastic. This will generally relieve the patient from all further trouble, especially if instructions are given to keep the bowels well open.

THE EXTRACTION OF PERMANENT TEETH.—Before extraction is commenced a few precautions are necessary. The instruments should be thoroughly clean, and disinfected by dipping into a one-in-twenty solution of carbolic acid; or a good plan is to have a pot of boiling water always in readiness, into which the instruments should be dipped for a minute or two before using. This acts in two ways: (1) as a disinfectant, (2) and it warms the instruments ready for use. Warmth is exceedingly necessary for the comfort of the patient, as, if the instrument is thoroughly warmed, the patient will often scarcely feel the forceps going on to the root. On the other hand, if you apply a cold instrument to a sensitive periosteum, you will give the patient a shock and cause him to flinch, and so lessen your chances of extracting the tooth. After the extraction do not forget to press the walls of the socket together with your fingers, as by doing this you bring into position again any fragments of the alveolus which have been forced outwards in the extraction of the tooth, and which, if left, often give rise to inflammation and much pain by irritating the periosteum. Here I must add that we should not try to do too much at one sitting. Some patients will experience very much more pain than others, especially those who have suffered from rheumatic fever and nervous diseases. Then again, patients who are very good at the time will often suffer very severely afterwards from headache and exhaustion. Although we can scarcely lay down any rules for extracting, yet, perhaps, after all, there is something in the old rule of: "First grasp your tooth, then move it in its socket, and lastly take it out." To these fundamental laws I would like to add a few more. Always take the tooth in the direction of least resistance, or, in other words, in the direction in which you feel the alveolus giving way. Lower wisdom teeth are generally best taken inwards, as they are supported externally by a strong ridge; viz., the external oblique line. Upper molars, on the other hand, will move in the outward direction most easily, as their long palatine fang acts as a buttress against inward movement, and the alveolus on the outer side is much thinner.

When the outer or inner surface of a molar tooth alone is standing, it will often be advisable to cut off the standing portion, in order to let the stump forceps go well on to the root. In extracting molar teeth with divergent roots, the dividing forceps are sometimes necessary. Having got the forceps well on, an attempt may be made to extract the tooth before crushing, as teeth will sometimes come out better with the dividing forceps than with the others. When the teeth are badly decayed, some operators use a spear-headed elevator, which they thrust in between the roots, and so loosen them for the stump forceps. Among the difficult teeth to extract may be mentioned those most delightfully lopsided molars described by Mr. Pearsall as oblique-rooted teeth, from which molar forceps invariably slip. They are, I think, best taken with a large pair of root forceps. Teeth with honeycombed crowns are also often very difficult to extract, owing to the immense length of their roots and the firmness with which they are implanted.

THE ELEVATOR.—The most useful forms are the straight oval shaped and the curved elevators, but some operators prefer the spear-headed form of straight elevator. The straight elevator is most useful in the extraction of the lower wisdom teeth when the molar in front is standing. For upper wisdoms it should not be used on account of the liability of fracturing the tuberosity and pterygoid process. It should be carefully guarded with the forefinger, when introduced into the mouth, and thrust deeply into the socket, between the wisdom and the adjacent tooth. The elevator should then be turned on its long axis, so that its lower edge cuts into the root of the tooth and raises it from its socket. The handle may then be drawn slightly forward and downwards. The thumb or finger of the left hand should at the same time be placed at the back of the tooth to prevent it going down the throat. To rotate the elevator on its long axis a fairly large handle is necessary, as little leverage is obtained. Great care is needed in moving the handle of the elevator much in the forward direction, as is sometimes practiced, for then the leverage is so great that there is danger of fracturing the adjacent tooth, or even the jaw itself. The curved elevator is most useful in extracting the roots of a first or second lower molar, especially the posterior root when the anterior has been removed with forceps. The elevator should be thrust into the socket behind the posterior root, and then the root can be turned out into the empty socket, the natural curvature of the root being in a suitable direction for it. I cannot agree with the plan of putting the elevator into the empty socket and bringing away the septum with the root, as I think you require much more force, and the parts become so bruised that you often get trouble afterwards.

Much more could be said of the complications of extraction, but time will not permit me to say more now. I hope, however, they will form the subject of another paper, together with hemorrhage and pain after extraction, which I have purposely left out. In conclusion, I must thank you all for the kind attention you have given me this evening, and I hope the discussion may prove more interesting.—*London Dental Record.*

